# Understanding structural distortions in hybrid layered perovskites with the *n* = 1 Ruddlesden–Popper structure

**DOI:** 10.1107/S2052252523003743

**Published:** 2023-06-13

**Authors:** Tianyu Liu, Noah P. Holzapfel, Patrick M. Woodward

**Affiliations:** a Department of Chemistry and Biochemistry, The Ohio State University, 100 West 18th Avenue, Columbus, OH 43210, USA; Formby, Liverpool, United Kingdom

**Keywords:** perovskites, symmetry mode analysis, organic–inorganic hybrid materials, Ruddlesden–Popper structure

## Abstract

A symmetry mode analysis yields 47 symmetrically distinct patterns of octahedral tilting in hybrid organic–inorganic layered perovskites that adopt the *n* = 1 Ruddlesden–Popper (RP) structure. The crystal structures of compounds belonging to this family are compared with the predictions of the symmetry analysis. Approximately 88% of the 140 unique structures have symmetries that agree with those expected based on octahedral tilting alone, while the remaining compounds have additional structural features that further lower the symmetry, such as asymmetric packing of bulky organic cations, distortions of metal-centered octahedra or a shift of the inorganic layers that deviates from the *a*/2 + *b*/2 shift associated with the RP structure. The structures of real compounds are heterogeneously distributed amongst the various tilt systems, with only 9 of the 47 tilt systems represented. No examples of in-phase ψ-tilts about the *a* and/or *b* axes of the undistorted parent structure were found, while at the other extreme ∼66% of the known structures possess a combination of out-of-phase ϕ-tilts about the *a* and/or *b* axes and θ-tilts (rotations) about the *c* axis. The latter combination leads to favorable hydrogen bonding interactions that accommodate the chemically inequivalent halide ions within the inorganic layers. In some compounds, primarily those that contain either Pb^2+^ or Sn^2+^, favorable hydrogen bonding interactions can also be achieved by distortions of the octahedra in combination with θ-tilts.

## Abstract 2

Structural distortions in hybrid organic–inorganic layered halide perovskites with the Ruddlesden–Popper structure are classified into 47 different patterns of octahedral tilting via symmetry mode analysis. A survey of known compounds shows that tilt systems with both out-of-phase ϕ-tilts about the *a* and/or *b* axes and θ-tilts (rotations) about the *c* axis are favored over other patterns of octahedral tilting because this combination leads to favorable hydrogen bonding interactions between the organic cations and the inorganic layers.

## 1. Introduction

The past decade has seen a resurgence in the study of hybrid halide perovskites, driven in part by the demonstration that high efficiency, solution processable photovoltaic cells can be made from materials like (CH_3_NH_3_)PbI_3_ (Stranks *et al.*, 2013 ▸; Baikie *et al.*, 2013 ▸; Frost *et al.*, 2014 ▸). The intense and sustained study of this family of materials has led to the discovery of many properties that are of interest for applications. Examples include photoluminescence (Stoumpos *et al.*, 2013 ▸; Majher *et al.*, 2019 ▸; Gray *et al.*, 2019 ▸), electroluminescence (Lin *et al.*, 2018 ▸), ferroelectricity (Liao *et al.*, 2015 ▸), low-dimensional magnetism (Asensio *et al.*, 2022 ▸) and colossal barocaloric effects (Li *et al.*, 2021 ▸; Seo *et al.*, 2022 ▸), among others.

**Figure 1:**
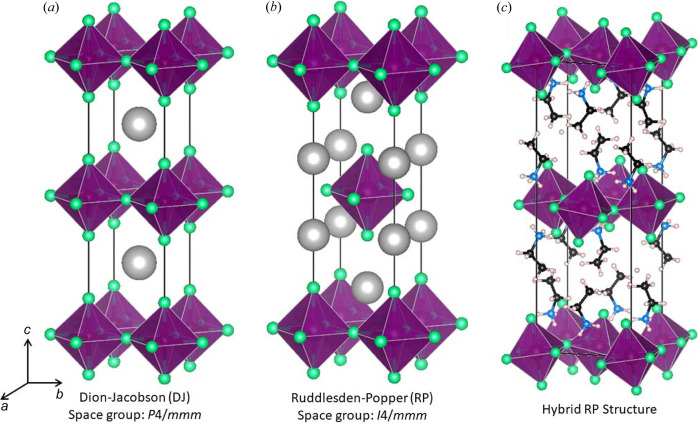
(*a*) Undistorted *n* = 1 DJ structure type. (*b*) Undistorted *n* = 1 RP structure type. (*c*) Hybrid layered perovskite with the *n* = 1 RP structure that exhibits octahedral tilting. For the DJ structure, two unit cells in the *c* direction are shown for ease of comparison.

Compositions within the 3D perovskite framework are limited to those containing relatively small organic cations, like methyl­ammonium and formamidinium. This restriction is lifted in 2D-layered perovskites where the octahedral connectivity in one direction is broken, leading to a vast family of hybrid layered perovskites. Broadly speaking, these can be further categorized into two groups. Those that fall into the Dion–Jacobson (DJ) family, where the inorganic layers stack in such a way that the octahedra line up on top of one another when viewed perpendicular to the layers [Fig. 1 ▸(*a*)], and those that fall into the Ruddlesden–Popper (RP) family, where the layers are offset by *a*/2 + *b*/2, so that the octahedra in each layer sit over the cavities in the adjacent layers [Fig. 1 ▸(*b*)]. Amongst all-inorganic compositions with layers one octahedron thick, RP phases have twice as many large ‘*A*-site’ cations as octahedra, giving a stoichiometry of *A*_2_ *BX*_4_, whereas the DJ phases have a stoichiometry of *ABX*_4_. However, in hybrid phases, the patterns of layer stacking are such that it is not always possible to classify compounds as belonging to the DJ or RP families from their composition alone. The structural implications of layer-stacking patterns, including layer shifts intermediate between the DJ and RP phases, are discussed at length in a recent paper by McNulty & Lightfoot (2021 ▸).

The prevalence of rotations or tilts of essentially rigid octahedra among 3D perovskites has long been appreciated (Glazer, 1972 ▸; Woodward, 1997*a* ▸,*b* ▸; Howard & Stokes, 1998 ▸; Stokes *et al.*, 2002 ▸; Howard *et al.*, 2003 ▸). Octahedral tilting distortions can have a dramatic impact on the physical properties of 3D inorganic perovskites and these distortions are often used to fine-tune the properties of both oxide and halide perovskites (Hwang *et al.*, 1995 ▸; Mahesh *et al.*, 1995 ▸; Attfield, 1998 ▸; Linaburg *et al.*, 2017 ▸). By and large, this approach to materials design has not been widely applied to hybrid layered perovskites [Fig. 1 ▸(*c*)], even though octahedral tilting distortions appear to be ubiquitous amongst these compounds (McNulty & Lightfoot, 2021 ▸). Nevertheless, there is every reason to believe that the optical, electrical and magnetic properties of hybrid layered perovskites can be tuned through the control of octahedral tilting distortions in a manner similar to that used for 3D oxide perovskites. To rationally design materials optimized for applications it is critical to understand the forces that drive octahedral tilting distortions. A detailed understanding of the crystal chemistry becomes even more important for phenomena that only emerge for specific structural distortions, like ferroelectricity.

In the first part of this paper, symmetry mode analysis is used to determine the space groups and unit cells associated with various patterns of octahedral tilting. To retain a manageable scope, the analysis is limited to RP phases with inorganic layers a single octahedron thick (*n* = 1 RP phases). The results for tilts/rotations perpendicular to the layers (θ-tilts) and/or out-of-phase tilts within the layers (ϕ-tilts) largely agree with the earlier analysis of Hatch *et al.* (1989 ▸), although some important differences are noted. Next, the analysis is extended to encompass in-phase tilts within the layers (ψ-tilts), which have not previously been considered. The results of the symmetry mode analysis are then compared with a tabulation of the crystal structures of known hybrid *n* = 1 RP phases. This comparison can help to establish the extent to which octahedral tilting distortions alone can be used to predict the symmetries of distorted structures, and to determine the patterns of octahedral tilting that are most common. Finally, hydrogen bonding interactions between the organic cations and inorganic layers are examined to better understand how they drive octahedral tilting distortions in hybrid layered perovskites. By going beyond classification and focusing on the crystal chemistry that drives these distortions it is hoped that this study will advance the ability of scientists to rationally design hybrid layered perovskites with useful physical properties.

## 2. Notation for describing octahedral tilting in *n* = 1 RP phases

There are two notations for describing octahedral tilting in perovskites, one developed by Glazer (1972 ▸) and the other by Alexandrov (1987 ▸). Glazer’s notation is widely used for 3D perovskites, but it has some limitations when applied to layered perovskites with the RP structure. Because the undistorted parent structure is tetragonal (*I*4/*mmm*) rather than cubic (*Pm* 3 *m*), the symmetry consequences of tilting about the *c* axis are different than tilting about the *a* and *b* axes. More importantly, the offset of *a*/2 + *b*/2 from one layer to the next means that the octahedra in adjacent layers are not aligned on top of one another and therefore one cannot speak of in-phase or out-of-phase tilting about the *c* axis, at least not for *n* = 1 RP phases. For these reasons the notation developed by Alexandrov is used throughout this paper.

**Figure 2:**
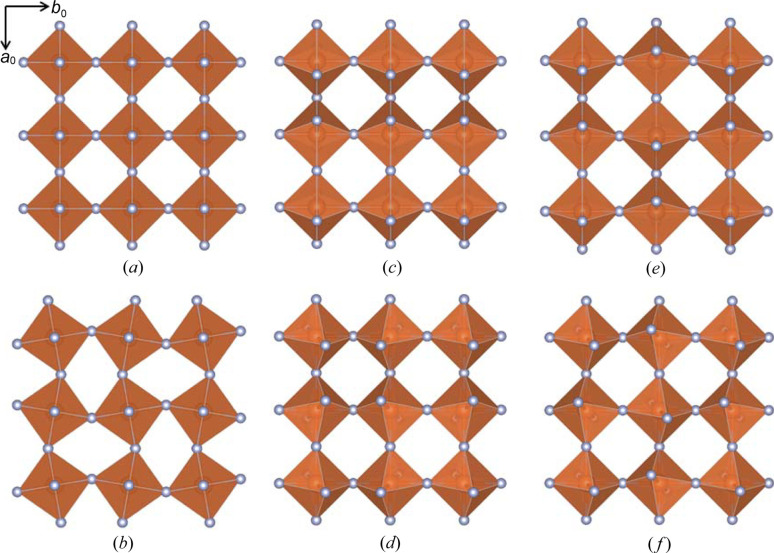
Types of octahedral tilting in layers of linked octahedra: (*a*) no tilting, (*b*) (00θ), (*c*) (0ψ0), (*d*) (ψψ0), (*e*) (0ϕ0) and (*f*) (ϕϕ0).

Fig. 2 ▸(*a*) shows an undistorted inorganic layer from a hybrid *n* = 1 RP phase. Rotations of the octahedra about the *c* axis of the tetragonal parent structure are denoted by the Greek letter θ and are illustrated in Fig. 2 ▸(*b*). Rotations about the *a* and/or *b* axes can be either in-phase or out-of-phase and are represented by the Greek letters ψ and ϕ, respectively. Fig. 2 ▸(*c*) illustrates in-phase ψ-tilts around the *b* axis, denoted (0ψ0). Simultaneous ψ tilts around both *a* and *b* are also possible, as illustrated by the (ψψ0) tilt system in Fig. 2 ▸(*d*). If there are tilts about two axes, but of different magnitudes, subscripts are used to signify their inequivalence (ψ_1_ψ_2_0). The same convention is used to describe out-of-phase ϕ-tilts. Examples of layers with (0ϕ0) and (ϕϕ0) tilting are illustrated in Figs. 2 ▸(*e*) and 2 ▸(*f*), respectively.

Because the octahedra in different layers are not connected, the tilting pattern in a given layer is, in principle, independent of the tilting in neighboring layers. To account for this, the tilting in each layer must be specified, for example, (ϕ00) tilting in layer 1 and (ϕϕθ) tilting in layer 2 would be written as (ϕ00)/(ϕϕθ). More complicated schemes where more than two layers are needed to capture the periodicity of the tilts are uncommon and therefore not considered in this analysis. In practice, the tilts are generally the same from one layer to the next; however, the directions of the tilts can differ from layer to layer, and this can have symmetry consequences, as discussed below.

To compare the direction of tilting in alternating layers, we compare the octahedron at the origin of the parent *I*4/*mmm* cell in one layer to the octahedron at the body center in the next layer. If these two octahedra tilt in the opposite direction it is written as either 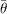 , 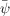 or 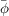 . In some cases, changing the direction of a tilt in layer 2 may not lead to a distinct tilt system. However, there are examples, such as (ϕϕθ)/(ϕϕθ) and (ϕϕθ)/(ϕϕ 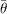 ), where changing the direction of one or more rotations in the second layer (but keeping the magnitude the same) does lead to a structure with symmetry that is distinct from other tilt systems.

## 3. Symmetry mode analysis

Aleksandrov and co-workers originally analyzed octahedral tilting in the *A*_2_ *BX*_4_ crystal structure through a direct crystallographic approach (Aleksandrov, 1987 ▸; Aleksandrov *et al.*, 1987*a* ▸). Their method consisted of physically depicting the movement of atoms caused by different variations of tilting and using this depiction to determine the symmetry elements present. Once the symmetry elements had been identified, a space group assignment could be made. Hatch *et al.* (1989 ▸) implemented a more systematic method for obtaining subgroups resulting from a single distortion. This analysis was carried out using a computational program that incorporated Landau’s theory of continuous phase transitions. Campbell *et al.* (2006 ▸) further developed this computer program into the *ISODISTORT* software suite, which can be used to explore the structural distortion modes of crystalline materials from a parent structure type. Here we use *ISODISTORT* to revisit the earlier analysis and expand it to include in-phase ψ tilts.

To determine the effects of various types of octahedral tilting on the *n* = 1 RP structure we use the crystal structure of K_2_NiF_4_ as the archetype or parent structure (Yeh *et al.*, 1993 ▸). This structure has tetragonal *I*4/*mmm* space group symmetry and unit-cell parameters *a*_0_ × *a*_0_ × *c*_0_ [Fig. 1 ▸(*b*)]. The subgroups determined by *ISODISTORT* were visualized with the ISOVIZ application which allowed the respective tilting scheme to be identified by visual inspection. The symmetry analysis was limited to structural distortion modes that can be described as rotations of the Ni-centered octahedra. Note that because hybrid layered perovskites with the DJ structure have a different parent structure, one with *P*4/*mmm* space group symmetry, the results of our analysis do not apply to DJ compounds or to compounds with layer shifts intermediate between RP and DJ phases. Interested readers are directed to earlier works by Aleksandrov *et al.* (1987*b* ▸), Aleksandrov & Bartolomé (2001 ▸) and McNulty & Lightfoot (2021 ▸) for a symmetry analysis of layered perovskites with the DJ structure. Note that the symmetry consequences of octahedral tilting in *A* *_n_*_+1_ *B* *_n_* *X*_3*n*+1_ RP phases differ depending on whether *n* is even or odd (Aleksandrov & Bartolome, 1994 ▸).

Hatch *et al.* (1989 ▸) previously identified the following irreducible representations (irreps) as being associated with the tilting of rigid octahedra: 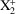 , 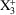 , 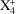 , 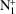 , P_4_ and P_5_, with the **k** vectors (1/2, 1/2, 0), (1/2, 0, 1/2) and (1/2, 1/2, 1/2) for X, N and P, respectively. The irreps are labeled with respect to the *I*4/*mmm* parent cell of the RP structure as depicted in Fig. 1 ▸(*b*). The symbols follow the notation of Miller & Love (1967 ▸), where the first term denotes the *k*-point of the Brillouin zone of the parent cell, and the superscript tells us whether the inversion center at the origin is retained (+) or lost (−). The distortions associated with the N and P irreps correspond to complex patterns of octahedral tilting that require more than two layers before repeating. With the exception of the P_4_ irrep, these types of octahedral tilting are rarely encountered, and therefore are not investigated further here. The P_4_ irrep produces a complex pattern of θ-tilts that repeats every four layers and leads to *I*4_1_/*acd* space group symmetry. As noted by Balachandran *et al.* (2014 ▸), this distortion is observed for several inorganic oxide compounds, including Ca_2_MnO_4_ and Sr_2_IrO_4_, but to the best of our knowledge is not observed among halide RP phases. Readers interested in such octahedral tilting patterns should revisit the original work (Hatch *et al.*, 1989 ▸). Also note that none of the six irreps listed above corresponds to in-phase ψ tilts. To expand the analysis to include ψ tilts, the incommensurate *k*-point, SM (*a*,0,0), must be included as one arm of the X *k*-point with *a* = 1/2.

**Figure 3:**
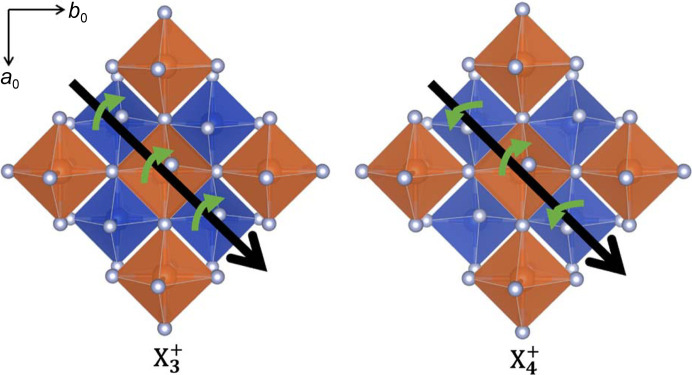
Top-down view of two distorted variants of the K_2_NiF_4_ structure showing the differences between 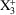 and 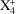 irreps that correspond to the order parameter (0, *a*). For clarity, the octahedra in the upper layer are depicted as orange octahedra, and those in the lower layer in blue. The 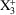 irrep leads to ϕ tilts around an axis parallel to [110] that have the same sense in all layers (left). The 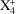 irrep produces the same ϕ tilts in the upper layer, but the direction of those tilts is reversed in adjacent layers (right).

Four irreps are found to be responsible for the tilts discussed in the previous section: 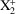 induces θ tilts, 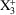 and 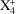 induce ϕ tilts, and SM3 induces ψ tilts. Examples of each are shown in Fig. S1 of the supporting information. The θ tilts can be described as rotations about the fourfold axes that run parallel to the *c* axis of the parent cell. The ψ tilts are in-phase rotations about axes that run parallel to either the *a* or the *b* axis of the parent cell, and the ϕ tilts are out-of-phase rotations about the axes that run along the face diagonals of the parent cell, either [110] or [110]. Though 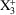 and 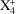 are both responsible for ϕ tilts, they lead to different tilts from one layer to the next, as shown in Fig. 3 ▸. If there is a clockwise tilt about [110] in layer 1 the 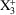 irrep will produce a clockwise tilt about the same axis in layer 2, whereas the 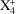 irrep will produce a counterclockwise tilt about this axis in layer 2.

Within the framework of Landau theory, the magnitude of each distortion is represented by an order parameter *g*. The order parameter identifies the invariant subspace containing all distortion vectors that possess the related distortion symmetry. Since the 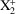 , 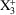 and 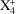 irreps are all 2D, two order parameters (*g*_1_ and *g*_2_) are associated with each irrep. For 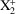 one order parameter signifies the magnitude of the rotation about the fourfold axis in layers 1 and 2 (the *c* axis of the parent cell). When the order parameter is (0, *a*) the octahedra in layers 1 and 2 rotate by the same magnitude, but the octahedron at the origin and the octahedron at the body center rotate in the opposite sense so that the tilt system is (00θ)/(00 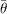 ). The second-order parameter also describes rotations about the fourfold axis, but the directions of the rotations alternate between adding to and opposing the rotations associated with the first-order parameter. Hence, when the 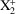 order parameter is (*a*, *a*) the rotations cancel in one layer and add in the second layer, giving the tilt system (000)/(00θ). When the magnitudes of the two order parameters are different (*a*, *b*) they produce rotations of different magnitudes in layers 1 and 2, giving the tilt system (00θ_1_)/(00θ_2_). From a symmetry perspective, the tilt system (00θ)/(00θ) is a special case of (00θ_1_)/(00θ_2_). In this structure, which has orthorhombic *Pbam* symmetry, there is no symmetry element that forces the rotations in one layer to be equal to those in the layers above and below. This finding differs from the earlier work of Hatch *et al.* (1989 ▸) where the symmetry associated with (00θ)/(00θ) tilting was erroneously listed as being identical to (00θ)/(00 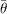 ).

As mentioned previously, the 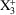 and 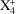 irreps are associated with ϕ tilts and correspond to rotations about face diagonals of the parent unit cell. When the order parameter is (0, *a*) the tilts are about [110] in each layer. For 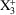 when the order parameter is (0, *a*) this leads to the (ϕϕ0)/(ϕϕ0) tilt system, while for the 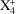 irrep the tilt system is (ϕϕ0)/( 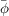 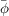 0). These two patterns of octahedral tilting are illustrated in Fig. 3 ▸. The order parameter (*a*, *a*) represents tilts about axes that run along either [110] or [110]. The net effect of this combination is a tilt about [100] in the first layer and [010] in the second layer, leading to the tilt system (ϕ00)/(0ϕ0) for 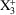 and (ϕ00)/(0 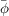 0) for 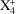 . If the order parameter is (*a*, *b*) the tilt systems that result are (ϕ_1_ϕ_2_0)/(ϕ_2_ϕ_1_0) and (ϕ_1_ϕ_2_0)/( 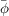 _2_ 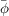 _1_0) for 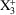 and 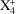 , respectively.

The results of our analysis for θ tilts, ϕ tilts and combinations of the two are given in Table 1 ▸. In addition to the nine tilt systems associated with a single irrep and described above, an additional sixteen tilt systems arise from the coupling of multiple irreps. The results of this analysis are in reasonably good agreement with the previous work of Hatch *et al.* (1989 ▸), but there are some differences. In addition to the differences in the tilt systems involving only θ-tilts discussed above, we obtain 25 unique tilt systems, an increase of 3 from the 22 previously reported. The additional tilt systems involve either coupling of 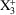 and 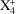 irreps or coupling of all three irreps. We also find that the tilt system previously reported as (ϕ_1_ϕ_2_θ)/( 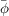 _2_ϕ_1_θ) should be classified as (ϕ_1_ϕ_2_θ)/( 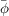 _2_ 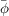 _1_θ) as shown in Fig. S2.

The symmetry analysis for in-phase ψ tilts described by the SM3 irrep, as well as those that result from the coupling of the SM3 and the 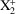 irreps are given in Table 2 ▸. Four order parameters are needed to describe the ψ tilting, two describe rotations about the *a* and *b* axes in the first layer and two about the same axes in the second layer. An additional five tilt systems that involve a combination of ψ-, ϕ- and θ-tilts are given in Table S1 of the supporting information. One interesting result from this study is that distortions involving ψ-tilts can produce noncentrosymmetric and polar space groups, whereas distortions involving only θ and ϕ tilts invariably result in centrosymmetric space groups. In retrospect, this result might have been anticipated from the fact that the 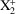 , 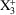 and 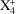 irreps all retain the inversion symmetry of the parent structure, hence the + superscript.

## 4. Observed patterns of octahedral tilting

The next task is to determine which patterns of octahedral tilting are most prevalent in real compounds. To do so the Inorganic Crystal Structural Database (ICSD) and Cambridge Structural Database (CSD) were surveyed to find halide variants of the *n* = 1 RP structure and classify their patterns of octahedral tilting. The tilt systems were determined by visual inspection of the reported crystal structures using the *VESTA3* software (Momma & Izumi, 2011 ▸), informed by the symmetry analysis discussed in the previous section.

First, the ICSD was surveyed for inorganic halide RP compounds. This resulted in 21 unique compositions (see Table S2). The compounds containing NH_4_^+^ are grouped with the inorganic phases because NH_4_^+^ is a spherical cation with no torsional degrees of freedom, and as such behaves more like an alkali cation than an organic cation (Lalancette *et al.*, 1972 ▸). At room temperature, 15 of the 21 compounds adopt the undistorted parent structure with *I*4/*mmm* symmetry. The only compounds that adopt a lower symmetry structure are those that contain a *B*-site cation prone to a Jahn–Teller (JT) distortion, either Cu^2+^ or Cr^2+^. A symmetry mode analysis of cooperative JT distortions in K_2_NiF_4_ compositions shows three symmetrically distinct patterns of distortion. If the elongated axis of the octahedron is oriented perpendicular to the layers the symmetry remains *I*4/*mmm*, but if the elongated axis is located within the layers the symmetry can be either *Cmce* or *Pbam*, depending on the directions of the distortions from one layer to the next. Interestingly, these two types of cooperative JT distortion are equivalent by symmetry to (00θ)/(00 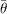 ) and (00θ)/(00θ) tilting, respectively. In all five compounds containing either Cu^2+^ or Cr^2+^, the cooperative JT distortion lowers the space group symmetry to *Cmce*. Though this symmetry permits (00θ)/(00 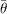 ) tilting, an inspection of the structure shows no sign of octahedral tilting. From this we conclude that, at room temperature, octahedral tilting distortions are generally not favorable in all-inorganic halides with the *n* = 1 RP structure. Low-temperature structural data are available only for K_2_MnF_4_ and (NH_4_)_2_MgF_4_. The former retains the undistorted *I*4/*mmm* structure down to 4 K, but the latter has *P*2_1_/*c* symmetry with the tilting scheme (ϕ_1_ϕ_2_θ)/(ϕ_2_ϕ_1_ 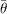 ) at 20 K. Note that an earlier study by Balachandran *et al.* (2014 ▸) found the undistorted *I*4/*mmm* structure is also the most common structure among oxides with the *n* = 1 RP structure.

A survey of hybrid compounds with organic cations separating the inorganic layers paints a very different picture. A search of both the CSD and ICSD revealed approximately 200 entries corresponding to layered hybrid organic–inorganic compounds with *A*_2_ *BX*_4_ stoichiometry and either Cl^–^, Br^–^ or I^–^ as the anion. The list can be culled down to 140 unique structures by eliminating: (1) isostructural entries with the same composition, (2) entries that have .cif files with flags that call into question their accuracy and (3) entries where the offset between layers differs from the *a*/2 + *b*/2 characteristic of the RP structure. The first criterion ensures that entries originating from variable-temperature studies where the same structure is observed at multiple different temperatures are counted only once. The second criterion culls questionable structure determinations. The third criterion eliminates entries that adopt the DJ structure or a structure that is intermediate between the RP and DJ structures. This is important because if the layer shift factor differs from *a*/2 + *b*/2 the symmetry of the parent space group is typically altered (McNulty & Lightfoot, 2021 ▸; Aleksandrov & Bartolomé, 2001 ▸). However, in those tilt systems with monoclinic or triclinic symmetry a layer shift factor different from *a*/2 + *b*/2 does not necessarily change the symmetry. This is most relevant for the (ϕϕθ)/(ϕϕ 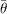 ) tilt system that gives rise to *P*2_1_/*c* symmetry. Aleksandrov *et al.* (1987*b* ▸) showed that (ϕϕθ) tilting in an *AMX*_4_ crystal with the DJ structure leads to the same unit cell and space group symmetry as (ϕϕθ)/(ϕϕ 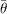 ) tilting in the RP structure. This explains why McNulty & Lightfoot (2021 ▸) found many examples of *n* = 1 DJ phases with this symmetry in their recent review. This is in contrast with tilt system (ϕϕθ)/(ϕϕθ), which leads to *Pbca* symmetry, where any deviation of the layer shift factor from *a*/2 + *b*/2 alters the symmetry. Entries removed due to irregular layer shifts tend to be most prevalent in compounds that have large and/or bulky organic cations. Details of the survey can be found in Tables S3 and S4.

Table 3 ▸ summarizes the frequencies with which each tilt system is observed among hybrid layered perovskite compounds found in the CCDC and ICSD. Of the 140 unique structures, 123 (88%) adopt a structure with symmetry that matches one of the tilt systems predicted in the previous section. The reasons why some compounds have symmetries that differ from the group theory predictions ultimately comes back to structural distortions that go beyond octahedral tilting. These will be discussed in the next section.

The first thing to note is that, unlike their all-inorganic counterparts, the undistorted structure is only observed at high temperatures (*T* > 340 K) and then only for six compounds. This finding is not too surprising as the cavities where the organic cations reside in the parent structure contain a fourfold axis and two mirror planes (point symmetry 4*mm* or *C*_4*v*_), a combination of symmetry elements not found in any organic cation among the hybrid compounds surveyed. To realize the symmetry of the undistorted parent structure the position of the organic cation must be disordered, and dynamic motions favored at high temperatures are the most likely source of this disorder. If there were more high-temperature structural studies there might be more examples of the parent structure, though in many cases decomposition would likely occur before a temperature is reached where the *I*4/*mmm* parent structure is stabilized.

The second observation of note is that, of the 47 possible tilt systems, only 9 are observed experimentally, none of which involve in-phase ψ tilts. Of those 9 tilt systems (ϕϕθ)/(ϕϕ 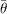 ) with monoclinic *P*2_1_/*c* symmetry and (ϕϕθ)/(ϕϕθ) with orthorhombic *Pbca* symmetry are the most common. In fact, tilt systems involving both ϕ- and θ-tilts account for ∼66% of the entries. There are seven examples of (ϕ_1_ϕ_2_θ)/(ϕ_1_ϕ_2_ 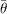 ) tilting, most of which contain large and/or bulky organic cations. It is likely that the packing of these large, often asymmetric organic cations causes the ϕ tilts within the layer to become inequivalent, lowering the space group symmetry from the monoclinic *P*2_1_/*c* associated with (ϕϕθ)/(ϕϕ 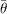 ) to the triclinic *P* 1 associated with (ϕ_1_ϕ_2_θ)/(ϕ_1_ϕ_2_ 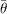 ) tilting.

The prevalence of tilt systems with both ϕ- or θ-tilts is even more pronounced at low temperatures. The point symmetry of the organic cation may play a role in this selection bias. The most common tilt systems involving only ϕ- or θ-tilts, namely (00θ)/(00 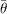 ), (ϕ00)/(0ϕ0) and (ϕϕ0)/(ϕϕ0), all have a mirror plane bis­ecting the cavity where the organic cation sits. In these structures the organic cations generally do not sit on the mirror plane and therefore exhibit some degree of disorder. In contrast, the glide planes and screw axes found in those tilt systems containing both ϕ- and θ-tilts do not impose symmetry constraints on the orientation or conformation of the organic cation, and in most but not all cases the organic cations are ordered.

Another interesting takeaway from Table 3 ▸ is the prevalence of deviations from the predicted symmetry for those compounds with (00θ)/(00 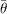 ) tilting. Over half of the entries that have only θ-tilts possess a space group symmetry lower than *Cmce*, in most cases the polar *Cmc*2_1_ space group. As discussed in the next section, this symmetry lowering is caused by a combination of orientational ordering of the organic cations and distortions of the octahedra.

**Figure 4:**
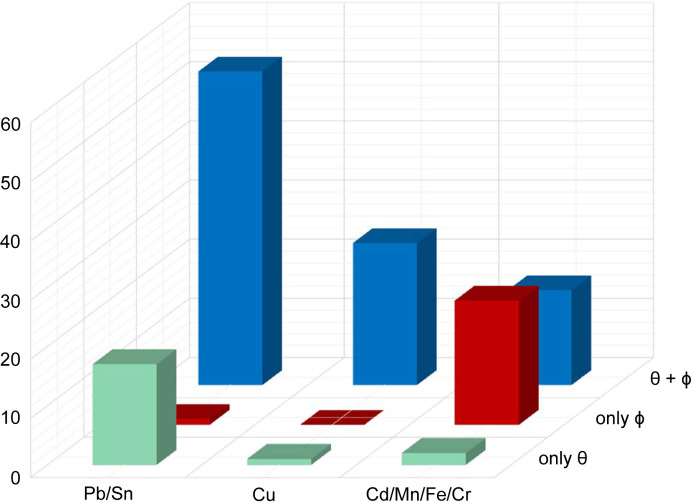
Prevalence of ϕ- and θ-tilts as a function of inorganic cation identity.

Fig. 4 ▸ shows that the types of tilts present depend in part on the composition of the inorganic layer. Examples of (00θ)/(00 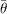 ) tilting are found predominantly in compounds where the inorganic cation is either Pb^2+^ or Sn^2+^, both of which are prone to stereoactive lone pair distortions. In contrast, examples with only ϕ-tilts are observed almost exclusively in compounds containing smaller inorganic cations such as Mn^2+^, Fe^2+^ and Cd^2+^ paired with the smaller chloride ion. The third group of compounds are those containing Cu^2+^ where a pronounced JT distortion leads to an ordered pattern of long and short bridging Cu—*X* bonds within the inorganic layers. As noted earlier, the cooperative JT distortion has symmetry consequences that are identical to θ-tilting. Hence, the combination of a cooperative JT distortion and ϕ-tilting would look very much like the combination of ϕ- and θ-tilting. Visual inspection of *A*_2_Cu*X*_4_ structures shows that in some cases θ-tilts are clearly present, while in other cases they are so small they could be ignored. Once the effects of the cooperative JT distortion are considered the patterns of octahedral tilting observed in *A*_2_Cu*X*_4_ compositions are similar to compounds containing Mn^2+^, Fe^2+^ and Cd^2+^.

It is also important to recognize that many compounds undergo changes in tilt system as a function of temperature. As such, temperature becomes an important variable when assessing the stabilities of competing tilt systems. Among compositions that contain divalent manganese, iron or cadmium it is common to observe both ϕ- and θ-tilts at low temperatures, but as the temperature increases the θ-tilts are lost (*i.e.* they become dynamic) leaving only ϕ-tilts.

## 5. Understanding tilt system preferences

The analysis in the previous section raises several questions. Why are some tilt systems favored over others? Why are ψ-tilts not observed? How do the identity and attributes of the inorganic cation, halide ion or organic cation affect the stabilities of competing tilt systems? What types of distortions are responsible for further reduction in symmetry and under what circumstances might we expect to observe them?

The structure-directing forces that are operative in hybrid *n* = 1 RP phases include polar-covalent bonding within the inorganic layers, noncovalent interactions (mostly dispersion forces) between organic cations, and hydrogen bonding between the organic cations and inorganic layers. The lack of octahedral tilting distortions in all-inorganic *n* = 1 RP phases suggests that bonding interactions within the inorganic layers are not the driving force behind octahedral tilting. Any impact of octahedral tilting on the packing of the organic cations would be a second-order effect. Therefore, we can assume that octahedral tilting distortions are largely driven by hydrogen bonding interactions between the ammonium head groups of the organic cations and the halide ions of the inorganic layer.

In the undistorted parent structure, the *A*-site cation sits on a site with 4*mm* (*C*_4*v*_) symmetry. It is surrounded by four terminal halide ions and four bridging halide ions. The presence of eight halide ions around the –NH_3_^+^ head group of the organic cation is not optimal for forming strong hydrogen bonds. Octahedral tilting distortions lower the symmetry, allowing some halide ions to move toward the ammonium head group while others move away. Tilt systems that create an environment where each hydrogen on the –NH_3_^+^ can form a strong hydrogen bond with a single halide ion will presumably be the most favorable.

**Figure 5:**
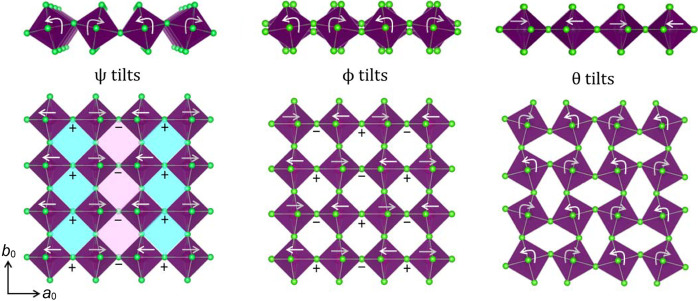
Halide ion displacements within a single *BX*_4_^2−^ layer with a ψ-tilt (left), ϕ-tilt (center) or θ-tilt (right), as viewed parallel (top) and perpendicular (bottom) to the layer. The + and − symbols indicate the movement of the bridging halide ions above and below the plane of the projection, respectively. The blue and pink shading shown in the lower left diagram highlights the chemical inequivalence of the *A*-sites resulting from ψ-tilts.

Fig. 5 ▸ shows the movements of the halide ions resulting from either ψ-, ϕ- or θ-tilts within a single layer. The first thing to note is that ψ-tilting creates two chemically inequivalent sites for the organic cations. For the sites shaded in blue, two of the four bridging halides move upward toward the *A*-site cation and all four terminal halides move away from the *A*-site cation. For the sites shaded in pink, the halide ions move in the opposite sense. Thus, we see that ψ-tilts create inequivalent environments for the organic cations, a violation of Pauling’s rule of parsimony (Pauling, 1929 ▸), which helps to explain why ψ-tilts are so rare.

The chemical and crystallographic inequivalence of the *A*-sites is not just for tilt systems with a (ψ00) layer. It is common to all tilt systems with in-phase ψ-tilts. Fig. 2 ▸(*d*) shows that this effect is even more pronounced for a layer with (ψψ0) tilting. A similar feature is known for 3D perovskites, where in-phase tilts about two or more axes lead to chemically inequivalent *A*-sites. In 3D perovskites, these patterns of tilting can generally only be stabilized by using *A*-site cations of different sizes and bonding preferences (Woodward, 1997*b* ▸). One such example is CaCu_3_Ti_4_O_12_, where *a* ^+^ *a* ^+^ *a* ^+^ tilting (in-phase tilting about all three axes) preserves the 12-fold coordination of the Ca^2+^ ion while the smaller Cu^2+^ ion adopts a fourfold square planar coordination environment (Subramanian *et al.*, 2000 ▸). It is not out of the question that a judicious choice of two or more organic cations might be used to a similar effect in layered hybrid perovskites. In fact, in-phase tilts are observed in two compositions containing a 1:1 mixture of different *A*-site cations – (methyl­ammonium, guanidinium)PbI_4_ and (caesium, guanidinium)PbBr_4_ – albeit with a 1/2*a* + 0*b* layer shift that is intermediate between an RP and a DJ phase (McNulty & Lightfoot, 2021 ▸).

**Figure 6:**
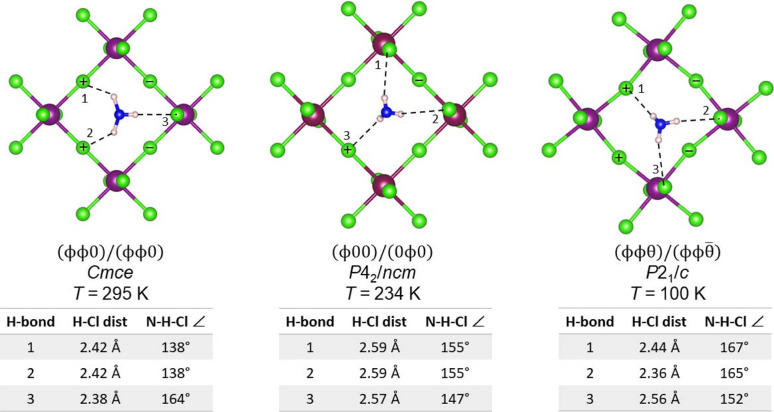
Hydrogen bonding in (CH_3_NH_3_)_2_CdCl_4_ at three different temperatures. The dashed lines represent the closest halide ion to each hydrogen of the –NH_3_^+^ head group. The + and − symbols indicate movements of the bridging chloride ions above or below the plane of the image, respectively. The CH_3_NH_3_^+^ cation shown is located above the CdCl_4_^2−^ layer.

Next, we turn our attention to tilt systems containing ϕ- and/or θ-tilts. The temperature-dependent structural evolution of (CH_3_NH_3_)_2_CdCl_4_ is illustrative. Not only does the small size of the methyl­ammonium cation minimize dispersion forces within the organic layer, but this compound also adopts three different tilt systems as a function of temperature: (ϕϕθ)/(ϕϕ 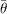 ) at 100 K, (ϕ00)/(0ϕ0) at 234 K and (ϕϕ0)/(ϕϕ0) at 295 K (Chapuis *et al.*, 1975 ▸). The hydrogen bonding interactions in each of these tilt systems are shown in Fig. 6 ▸. As the temperature is lowered and the effects of entropy are reduced, the enthalpy term, which contains a contribution from hydrogen bonding, makes an ever-larger contribution to the free energy. From this we can infer that the strength of the hydrogen bonding increases as the temperature is lowered: (ϕϕ0)/(ϕϕ0) < (ϕ00)/(0ϕ0) < (ϕϕθ)/(ϕϕ 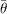 ). At first glance, this relative order is not obvious as the H—Cl distances are on average shortest at room temperature where (ϕϕ0)/(ϕϕ0) tilting is observed.

To better understand this order of the phase transitions in (CH_3_NH_3_)_2_CdCl_4_ we need to consider the hydrogen bonding interactions more carefully. Let us begin by putting the H—Cl distances and N—H—Cl angles into context. Steiner (1998 ▸) reviewed the distribution of hydrogen bond distances and angles in halide salts of various organic cations, including primary ammonium cations. He found that for ‘nearly linear’ hydrogen bonds, defined as those with N—H—Cl angles >140°, the average H—Cl distance was 2.247 (5) Å and nearly all of these bonds fell between 2.1 and 2.5 Å. In layered hybrid perovskites, the hydrogen bonds are on the longer side of this distribution because the chlorides form either one or two covalent bonds with cadmium and therefore have less bonding capacity than a free halide ion. However, based on Steiner’s distance criterion we could qualitatively assign three strong hydrogen bonds in (ϕϕ0)/(ϕϕ0), three weak hydrogen bonds in (ϕ00)/(0ϕ0) and two strong plus one weak hydrogen bond in (ϕϕθ)/(ϕϕ 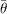 ). Although this analysis neglects the N—H—Cl angles, which are closer to linear in (ϕϕθ)/(ϕϕ 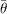 ) than they are in the other two tilt systems.

Next, we consider the bonding requirements of the chloride ions. In a simplistic model where the Cd—Cl bonds are all treated as equivalent, the +2 oxidation state of cadmium dictates a valence of 2/6 = 1/3 for each Cd—Cl bond. Therefore, the bridging halide ion is assigned 2 × (1/3) = 2/3, and the terminal halide ion is assigned 1 × (1/3) = 1/3 of its expected valence from bonding to cadmium. The unfulfilled bonding must come from hydrogen bonds. In practice, the terminal Cd—Cl bonds are somewhat shorter [2.537 (4) Å] than the terminal Cd—Cl bonds [2.644 (3) Å] at 295 K, but that does not invalidate the notion that the terminal chlorides must form either stronger or more numerous hydrogen bonds than the bridging chlorides.

We can be more quantitative if we calculate bond valence sums from the experimentally observed Cd—Cl bond distances (Brown, 2016 ▸). If we neglect any contribution from hydrogen bonds, the bond valence sum at the terminal chloride ion is 0.44, while the bond valence sum of the bridging chloride ion is 0.65. Similar bond valence sums are obtained for a series of Pb-containing *n* = 1 hybrid RP phases (see Fig. S3), where the terminal halides are found to have bond valence sums ranging from 0.36 to 0.44, and the bridging halides values ranging from 0.71 to 0.85.

Returning to Fig. 6 ▸, notice that (ϕϕ0)/(ϕϕ0) tilting leads to one hydrogen bond with a terminal halide and two with bridging halides. It follows that in this structure each terminal halide forms one hydrogen bond, while each bridging halide forms two hydrogen bonds. This configuration, referred to as the bridging configuration by Mitzi (2007 ▸), is at odds with the bonding requirements of the halide ions within the inorganic layer. In contrast, the patterns of hydrogen bonding observed in (ϕ00)/(0ϕ0) and (ϕϕθ)/(ϕϕ 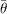 ) have the opposite configuration, two bonds to terminal halides and one to a bridging chloride. This configuration, dubbed the terminal configuration, is better able to meet the bonding requirements of the chloride ions in the inorganic layer, which may help to explain why these tilt systems are more stable. Of the two, (ϕϕθ)/(ϕϕ 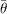 ) has hydrogen bonds that are both shorter and closer to the ideal linear geometry. This allows us to rationalize why the hydrogen bonding observed in (ϕϕθ)/(ϕϕ 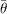 ) is more favorable than realized in the other two tilt systems.

Of the tilt systems observed in real compounds only (ϕϕ0)/(ϕϕ0) leads to the less favorable bridging configuration. Yet there are no fewer than seven different *A*_2_Cd*X*_4_ compositions that have (ϕϕ0)/(ϕϕ0) tilting. Examples are found for *n* = 1 RP phases with iron and manganese as well. However, in those cases where diffraction studies have been carried out at low temperatures, the (ϕϕ0)/(ϕϕ0) pattern usually transforms to a tilt system with both ϕ- and θ-tilting on cooling. Also note that for organic cations larger than methyl­ammonium (CH_3_NH_3_^+^) the symmetry elements associated with (ϕϕ0)/(ϕϕ0) tilting lead to disorder in the positions of the hydro­carbon part of the organic cation. Presumably, the configurations and/or orientations of the organic cation are dynamic at high temperatures and couple to dynamic θ-tilts of the inorganic layer. At low temperatures these lattice vibrations freeze out, leading to a transition into a tilt system with both ϕ- and θ-tilts.

Finally, let us consider the competition between the two most common tilt systems: (ϕϕθ)/(ϕϕ 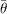 ) which leads to monoclinic *P*2_1_/*c* symmetry and (ϕϕθ)/(ϕϕθ) which leads to orthorhombic *Pbca* symmetry. Because the tilting in any one layer is of the (ϕϕθ) type in both tilt systems, very similar patterns of hydrogen bonding emerge. However, the alternation in the direction of the θ-tilts observed in the *Pbca* structure will lead to slightly different orientations of the organic cations than those observed in the *P*2_1_/*c* structure. This suggests that dispersion forces between organic cations play a key role in the competition between these two tilt systems. Evidence to support this hypothesis can be found among the behavior of *n* = 1 lead iodide RP phases containing linear alkyl ammonium cations, [H(CH_2_)_*n*_NH_3_]_2_PbI_4_ (Billing & Lemmerer, 2007 ▸, 2008 ▸; Lemmerer & Billing, 2012 ▸). For compounds with *n* ≤ 10, a transition from (ϕϕθ)/(ϕϕ 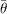 ) tilting to (ϕϕθ)/(ϕϕθ) tilting occurs on heating. In contrast, those compositions with even longer alkyl ammonium cations (*n* = 12, 14, 16 and 18) show the opposite behavior (ϕϕθ)/(ϕϕθ) is more stable at low temperatures and (ϕϕθ)/(ϕϕ 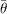 ) at high temperatures. The alkyl­ammonium copper chloride series [H(CH_2_)_*n*_NH_3_]_2_CuCl_4_ also shows an interesting dependence on the length of the organic cation. At room temperature (ϕϕθ)/(ϕϕ 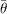 ) tilting and monoclinic symmetry are observed when the organic cation is methyl­ammonium, whereas (ϕϕθ)/(ϕϕθ) tilting and orthorhombic symmetry is observed for ethyl­ammonium copper chloride.

## 6. Distortions other than octahedral tilting

**Figure 7:**
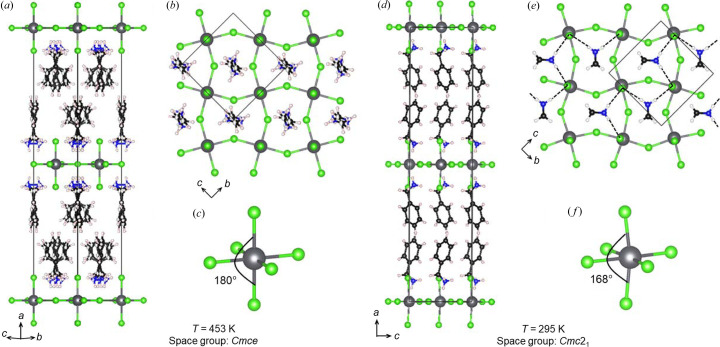
Crystal structures of (C_6_H_5_CH_2_NH_3_)_2_PbCl_4_ at 453 K (left) and 295 K (right). Views of the high-temperature centrosymmetric structure include (*a*) the unit cell as viewed parallel to the inorganic layers; (*b*) a top-down view of a single inorganic layer, where the benzyl groups of the organic cation have been omitted for clarity; and (*c*) the Pb-centered octahedron. Comparable views of the low-temperature polar structure are shown in (*d*), (*e*) and (*f*), with strong hydrogen bonds denoted by dashed lines in (*d*). The axes shown refer to the standard setting of these two space groups.

The relative abundance of hybrid *n* = 1 RP phases with (00θ)/(00 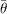 ) tilting observed in Table 3 ▸ is somewhat surprising. As we can see from Fig. 5 ▸ the positions of the terminal halide ions are not greatly affected by θ-tilting and remain equidistant from the center of the cavity where the −NH_3_^+^ group sits. In this geometry, how can strong hydrogen bonds to the terminal halides form, as needed to satisfy their bonding requirements? A closer look at the entries in Tables S3 and S5 shows that many compositions with (00θ)/(00 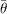 ) tilting undergo a transition from *Cmce* to *Cmc*2_1_ on cooling. The crystal structures of one such example, (BzA)_2_PbCl_4_ (BzA = benzyl­ammonium, C_6_H_5_CH_2_NH_3_^+^), are shown at temperatures above and below this phase transition in Fig. 7 ▸ (Liao *et al.*, 2015 ▸). On the left-hand side, we see the structure at 453 K in the high-temperature *Cmce* symmetry. The absence of ϕ-tilts and the presence of large θ-tilts can be clearly observed, along with the disorder of the BzA cations. Ignoring the disorder, the shortest H—Cl distance is 2.59 Å and the shortest distance between a hydrogen and a terminal chloride is 2.68 Å. In the low-temperature *Cmc*2_1_ structure, the BzA cations adopt an ordered pattern and the terminal chloride ions shift toward two of the four neighboring –NH_3_^+^ groups. This leads to a pattern of strong hydrogen bonds with H—Cl distances of 2.33 to 2.35 Å and N—H—Cl angles ranging from 163 to 172° shown in Fig. 7 ▸(*e*). These hydrogen bonds are comparable in strength to the strongest bonds observed in the low-temperature structure of (CH_3_NH_3_)_2_CdCl_4_ discussed above. The third hydrogen atom on the ammonium head group is equidistant between the two bridging chloride ions, forming a weaker bifurcated hydrogen bond with an H—Cl distance of 2.63 Å to both bridging chlorides.

Interestingly, the terminal chloride ions above and below each Pb^2+^ ion shift in the same direction, which destroys the inversion center present in the high-temperature structure and bends the Cl—Pb—Cl bond angle from the linear 180° observed in the high-temperature structure to 168° [Figs. 7 ▸(*c*) and 7 ▸(*f*)]. As shown in Fig. 7 ▸(*e*), half of the positively charged –NH_3_^+^ groups point in the direction parallel to [011] and the other half in the direction parallel to [011]. When combined the net result is a permanent dipole moment along the *c* axis, which is the polar axis for the *Cmc*2_1_ space group. (BzA)_2_PbCl_4_ not only adopts a polar structure below 436 K, the polar axis can be reversed with an electric field making it a ferroelectric. It is notable that nearly all hybrid *n* = 1 RP phases showing this phase transition contain either Pb^2+^ or Sn^2+^. The 5*s* ^2^/6*s* ^2^ electron configuration of these ions may play a role in stabilizing the deformations of the octahedra that accompany the orientational ordering of the organic cations. A subtle distortion is also observed in the pattern of bonds in the plane defined by lead and the bridging chloride ions, with two Pb—Cl bond distances of 2.84 Å and two distances of 2.89 Å in the low-temperature *Cmc*2_1_ structure. However, this type of distortion is also observed in Cd-containing RP phases with benzyl­ammonium and cyclo­hexyl­ammonium cations, so the attributes of the organic cation also plays a role.

Symmetry lowering due to orientational ordering of the organic cations is not exclusive to compounds containing Pb^2+^ or Sn^2+^ and (00θ)/(00 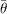 ) tilting (see Table S5). Both (IBA)_2_­CdBr_4_ [IBA = iso­butyl­ammonium, (CH_3_)_2_CH_2_CH_2_NH_3_^+^] and (IPA)_2_CdCl_4_ [IPA = iso­propyl­ammonium, (CH_3_)_2_CH_2_CH_2_­CH_2_NH_3_^+^] have room-temperature structures with (ϕϕ0)/(ϕϕ0) tilting and *Cmce* symmetry. In both compounds, a transition to the polar space group *C*2*ce* (or non-standard setting *Aea*2) driven by the orientational ordering of the organic cations occurs on cooling.

Up to this point, we have largely focused on distortions of the inorganic layers and how those distortions impact hydrogen bonding, but the identity and attributes of the organic cation and the halide anion also play a role in determining the relative stability of competing structural distortions. The (Bz*A*)_2_PbCl_4_ structure discussed above undergoes a *Cmce* to *Cmc*2_1_ phase transition at ∼436 K (Liao *et al.*, 2015 ▸). At room temperature (Bz*A*)_2_PbBr_4_ also possesses *Cmc*2_1_ symmetry (Du *et al.*, 2017 ▸), whereas (Bz*A*)_2_PbI_4_ adopts a structure with (ϕϕθ)/(ϕϕ 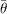 ) tilting and *Pbca* symmetry at room temperature (Papavassiliou *et al.*, 1999 ▸). This suggests that the stabilizing distortion of the *Cmc*2_1_ structure becomes less favorable as the halide ion becomes larger and less electronegative. The prevalence of (00θ)/(00 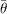 ) tilting among *A*_2_Pb*X*_4_ and *A*_2_Sn*X*_4_ compositions seems to be limited primarily to compositions containing aromatic or cyclic ammonium cations, whereas those containing linear alkyl ammonium cations tend to exhibit tilt systems with both ϕ- and θ-tilts. From this observation, one can infer that the packing of the organic cations plays a role in determining the most favorable tilt system.

## 7. Conclusions

Using *ISODISTORT* the symmetry consequences of octahedral tilting in hybrid layered perovskites with the *n* = 1 RP structure have been analyzed. In total, 25 different patterns of octahedral tilting are obtained by combining tilts/rotations about the *c* axis (θ-tilts) of the *I*4/*mmm* parent structure and out-of-phase tilts about the *a* and *b* axes of the parent structure (ϕ-tilts). An additional 22 patterns of tilting are obtained when in-phase tilts (ψ-tilts) about the *a* and *b* axes are considered. The predicted structures have been compared with the structures of hybrid halide perovskites found in the CSD and ICSD. Of the 140 unique structures found in these databases, 123 (88%) adopt structures that are consistent with the predictions of the symmetry analysis. Of the 47 possible tilt systems, only 9 are observed experimentally (excluding the undistorted parent structure), none of which involve ψ tilts. In contrast, distortions involving both ϕ- and θ-tilts are common, accounting for ∼66% of the entries. The combination of ϕ- and θ-tilts is stabilized by favorable hydrogen bonding interactions between the organic cations and the inorganic layers. For the remaining 12% of the structures, the symmetry is further lowered by effects such as orientational ordering of the organic cations and/or distortions of the octahedra. Distortions that go beyond octahedral tilting are particularly prominent in compounds with (00θ)/(00 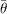 ) tilting that contain either Pb^2+^ or Sn^2+^ ions. In those compounds, distortions of the octahedra offer an alternate route to the formation of strong hydrogen bonding interactions.

## Supplementary Material

Supporting figures and tables. DOI: 10.1107/S2052252523003743/lt5057sup1.pdf

## Figures and Tables

**Table 1:** Space group assignments and tilting schemes that arise from the combination of θ and/or ϕ tilts for *n* = 1 RP phases

No.	Tilts		Space group	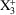	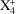	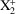	Basis	Origin
	First layer	Second layer						
1	00θ	00 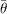	*Cmce*	(0, 0)	(0, 0)	(0, *a*)	(0,0,1), (1,1,0), (1,1,0)	(0,0,0)
2	000	00θ	*P*4/*mbm*	(0, 0)	(0, 0)	(*a*, *a*)	(1,1,0), (1,1,0), (0,0,1)	(1/2,1/2,0)
3	00θ_1_	00θ_2_	*Pbam*	(0, 0)	(0, 0)	(*a*, *b*)	(1,1,0), (1,1,0), (0,0,1)	(0,0,0)
4	ϕϕ0	ϕϕ0	*Cmce*	(0, *a*)	(0, 0)	(0, 0)	(1,1,0), (0,0,1), (1,1,0)	(0,0,0)
5	ϕ00	0ϕ0	*P*4_2_/*ncm*	(*a*, *a*)	(0, 0)	(0, 0)	(1,1,0), (1,1,0), (0,0,1)	(0,0,0)
6	ϕ_1_ϕ_2_0	ϕ_2_ϕ_1_0	*Pccn*	(*a*, *b*)	(0, 0)	(0, 0)	(1,1,0), (1,1,0), (0,0,1)	(0,0,0)
7	ϕϕ0	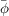 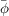 0	*Cccm*	(0, 0)	(*a*, 0)	(0, 0)	(0,0,1), (1,1,0), (1,1,0)	(−1/4,1/4,1/4)
8	ϕ00	0 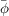 0	*P*4_2_/*nnm*	(0, 0)	(*a*, *a*)	(0, 0)	(1,1,0), (1,1,0), (0,0,1)	(1/2,1/2,0)
9	ϕ_1_ϕ_2_0	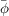 _2_ 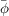 _1_0	*Pnnn*	(0, 0)	(*a*, *b*)	(0, 0)	(1,1,0), (1,1,0), (0,0,1)	(0,0,0)
10	ϕϕθ	ϕϕθ	*Pbca*	(0, *a*)	(0, 0)	(*b*, 0)	(0,0,1), (1,1,0), (1,1,0)	(0,0,0)
11	ϕϕθ	ϕϕ 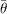	*P*2_1_/*c*	(0, *a*)	(0, 0)	(0, *b*)	(1/2,1/2,1/2), (1,1,0), (1,1,0)	(0,0,0)
12	ϕ_1_ϕ_2_θ	ϕ_2_ϕ_1_ 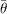	*P*2_1_/*c*	(*a*, *b*)	(0, 0)	(0, *c*)	(1,1,0), (1,1,0), (0,0,1)	(0,0,0)
13	ϕϕθ	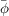 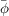 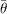	*Pccn*	(0, 0)	(*a*, 0)	(*b*, 0)	(0,0,1), (1,1,0), (1,1,0)	(0,0,0)
14	ϕϕθ	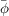 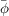 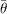	*C*2/*c*	(0, 0)	(*a*, 0)	(0, *b*)	(0,0,1), (1,1,0), (1,1,0)	(1/4,−1/4,−1/4)
15	ϕ_1_ϕ_2_θ	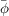 _2_ 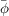 _1_ 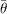	*P*2/*c*	(0, 0)	(*a*, *b*)	(0, *c*)	(1,1,0), (1,1,0), (1,1,1)	(0,0,0)
16	ϕ_1_ϕ_2_0	ϕ_1_ϕ_2_0	*C*2/*c*	(0, *a*)	(0, *b*)	(0, 0)	(1,1,0), (0,0,1), (1,1,0)	(1/4,1/4,−1/4)
17	ϕ_1_ϕ_1_0	ϕ_2_ϕ_2_0	*Pmna*	(0, *a*)	(*b*, 0)	(0, 0)	(1,1,0), (0,0,1), (1,1,0)	(0,0,0)
18	ϕ_1_00	0ϕ_2_0	*Cmma*	(*a*, *a*)	(*b*, *b*)	(0, 0)	(2,0,0), (0,2,0), (0,0,1)	(0,0,0)
19	ϕ_1_ϕ_2_0	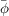 _2_ϕ_1_0	*P*4_2_/*n*	(*a*, *a*)	(*b*, −*b*)	(0, 0)	(1,1,0), (1,1,0), (0,0,1)	(1/2,−1/2,−1/2)
20	ϕ_1_ϕ_2_0	ϕ_3_ϕ_4_0	*P*2/*c*	(*a*, *b*)	(*c*, *d*)	(0, 0)	(1,1,0), (0,0,1), (0,2,0)	(0,0,0)
21	ϕ_1_ϕ_2_θ	ϕ_1_ϕ_2_θ	*P*2_1_/*c*	(0, *a*)	(0, *b*)	(*c*, 0)	(1,1,0), (0,0,1), (1,1,0)	(0,0,0)
22	ϕ_1_ϕ_2_θ	ϕ_1_ϕ_2_ 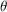	*P* 1	(0, *a*)	(0, *b*)	(0, *c*)	(1,1,0), (1,1,0), (1/2,1/2,−1/2)	(0,0,0)
23	ϕ_1_ϕ_1_θ_1_	ϕ_2_ϕ_2_θ_2_	*P*2_1_/*c*	(0, *a*)	(*b*, 0)	(*c*, *d*)	(0,0,1), (1,1,0), (1,1,0)	(0,0,0)
24	ϕ_1_00	0ϕ_2_θ	*C*2/*m*	(*a*, *a*)	(*b*, *b*)	(*c*, *c*)	(0,2,0), (2,0,0), (0,0,1)	(0,0,0)
25	ϕ_1_ϕ_2_θ_1_	ϕ_3_ϕ_4_θ_2_	*P* 1	(*a*, *b*)	(*c*, *d*)	(*e*, *f*)	(1,1,0), (1,1,0), (0,0,1)	(0,0,0)

**Table 2:** Space group assignments and tilting schemes that arise from the SM3 irrep or a combination of SM3 and 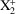 irreps

No.	Tilts		Space group	SM3	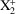	Basis	Origin
	First layer	Second layer					
26	0ψ0	000	*Pmma*	(*a*,0); (0,0)	(0, 0)	(2,0,0), (0,1,0), (0,0,1)	(0,0,0)
27	0ψ0	0 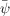 0	*Pnma*	(*a*,*a*); (0,0)	(0, 0)	(2,0,0), (0,1,0), (0,0,1)	(3/4,1/4,1/4)
28	ψψ0	000	*P*4/*nmm*	(*a*,0); (*a*,0)	(0, 0)	(2,0,0), (0,2,0), (0,0,1)	(0,0,0)
29	ψ00	0ψ0	*P*4_2_/*nmc*	(0,*a*); (*a*,0)	(0, 0)	(2,0,0), (0,2,0), (0,0,1)	(−3/2,0,−1/2)
30	ψψ0	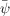 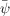 0	*Cmce*	(*a*,*a*); (*a*,*a*)	(0, 0)	(2,2,0), (2,2,0), (0,0,1)	(−1/4,7/4,1/4)
31	0ψ_1_0	0ψ_2_0	*Pmc*2_1_	(*a*,*b*); (0,0)	(0, 0)	(0,1,0), (0,0,1), (2,0,0)	(0,0,0)
32	ψ_1_ψ_2_0	000	*Pmmn*	(*a*,0); (*b*,0)	(0, 0)	(2,0,0), (0,2,0), (0,0,1)	(0,0,0)
33	ψ_1_00	0ψ_2_0	*Pmmn*	(0,*a*); (*b*,0)	(0, 0)	(2,0,0), (0,2,0), (0,0,1)	(−1/2,0,0)
34	ψ_1_ψ_1_0	ψ_2_ψ_2_0	*Abm*2	(*a*,*b*); (*a*,*b*)	(0, 0)	(0,0,1), (2,2,0),(2,2,0)	(0,1,0)
35	ψ_1_ψ_2_θ	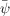 _1_ 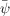 _2_θ	*P*2_1_/*c*	(*a*,*a*); (*b*,*b*)	(0, *c*)	(2,0,0), (0,0,1), (2,2,0)	(−5/4,3/4,1/4)
36	ψ_1_ψ_2_θ	ψ_1_ψ_2_ 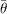	*Aba*2	(*a*,*b*); (*b*,*a*)	(*c*, 0)	(0,0,1), (2,2,0), (2,2,0)	(0,3/2,1/4)
37	0ψ0	00θ	*Pmna*	(*a*,0); (0,0)	(*b*, *b*)	(0,2,0), (0,0,1), (2,0,0)	(0,0,0)
38	ψψ0	00θ	*P*4/*n*	(*a*,0); (*a*,0)	(*b*, *b*)	(0,2,0), (2,0,0), (0,0,1)	(0,−1,0)
39	ψ_1_ψ_2_θ	000	*Pmmn*	(*a*,0); (*b*,0)	(*c*, −*c*)	(2,0,0), (0,2,0), (0,0,1)	(0,0,0)
40	ψ_1_ψ_2_θ	0ψ_3_0	*Pmn*2_1_	(*a*,*b*); (*c*,0)	(*d*, −*d*)	(0,2,0), (0,0,1), (2,0,0)	(0,1/2,0)
41	ψ_1_ψ_2_θ_1_	00θ_2_	*P*2/*c*	(*a*,0); (*b*,0)	(*c*, *d*)	(2,0,0), (0,0,1), (2,2,0)	(0,0,0)
42	ψ_1_ψ_2_θ_1_	ψ_3_ψ_4_θ_2_	*Pc*	(*a*,*b*); (*c*,*d*)	(*e*, *f*)	(2,0,0), (0,0,1), (2,2,0)	(0,0,0)

**Table 3:** Frequency of tilt systems observed in *A*_2_ *BX*_4_ hybrid halide perovskites The entries are divided into those that have the space group symmetry predicted by group theory and those that have lower symmetry.

Tilt system	Predicted space group symmetry	Entries that agree with the prediction	Entries with symmetry lower than predicted	Total entries
No tilts				
(000)/(000)	*I*4/*mmm*	6	0	6
Only θ tilts				
(00θ)/(00θ)	*Cmce*	8	11	19
Only ϕ tilts				
(ϕ00)/(0ϕ0)	*P*4_2_/*ncm*	4	0	4
(ϕϕ0)/(ϕϕ0)	*Cmce*	12	4	16
(ϕ_1_ϕ_2_0)/(ϕ_2_ϕ_1_0)	*Pccn*	3	0	3
Both θ and ϕ tilts				
(ϕϕθ)/(ϕϕθ)	*P*2_1_/*c*	41	1	42
(ϕϕθ)/(ϕϕθ)	*Pbca*	38	1	39
(ϕϕθ)/(ϕ ϕ θ)	*C*2/*c*	3	0	3
(ϕ_1_ϕ_2_θ)/(ϕ_1_ϕ_2_θ)	*P*2_1_/*c*	1	0	1
(ϕ_1_ϕ_2_θ)/(ϕ_1_ϕ_2_ θ)	*P* 1	7	0	7
