# Supplementary material for: Understanding structural distortions in hybrid layered perovskites with the n = 1 Ruddlesden–Popper structure
Source: IUCrJ. 2023 Jun 13;10(Pt 4):385–96. doi: 10.1107/S2052252523003743 (PMC10324490; doi:10.1107/S2052252523003743)
Supplement: Supplementary file 1 [file m-10-00385-sup1.pdf]

IUCrJ

Volume 10 (2023)

Supporting information for article:

Understanding structural distortions in hybrid layered perovskites with the n = 1 Ruddlesden-Popper structure

Tianyu Liu, Noah P. Holzapfel and Patrick M. Woodward

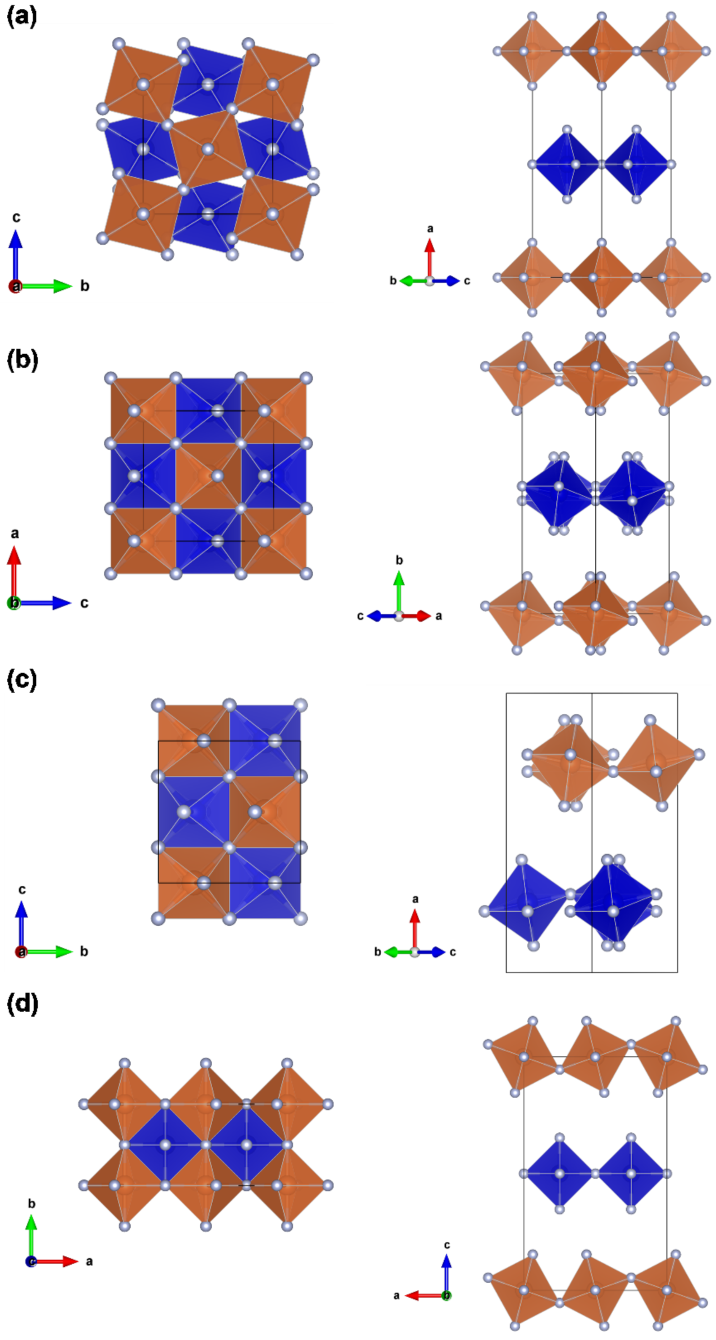

Figure S1: Examples of tilt systems from different views. The corresponding numbered results can be found in Table 1, Table 2, and Appendix A: (**a**) tilt system #1 $(00\theta)/(00\bar{\theta})$ with *Cmce* symmetry from irrep X_2^+; (**b**) tilt system #4 $(\phi\phi0)/(\phi\phi0)$ with *Cmce* symmetry from irrep X_3^+; (**c**) tilt system #7 $(\phi\phi0)/(\bar{\phi}\bar{\phi}0)$ with *Cccm* symmetry from irrep X_4^+; (**d**) tilt system #26 $(0\psi0)/(000)$ with *Pmma* symmetry from irrep SM3. The A-sites are removed for clarity and the octahedra are color coded to help differentiate layers.

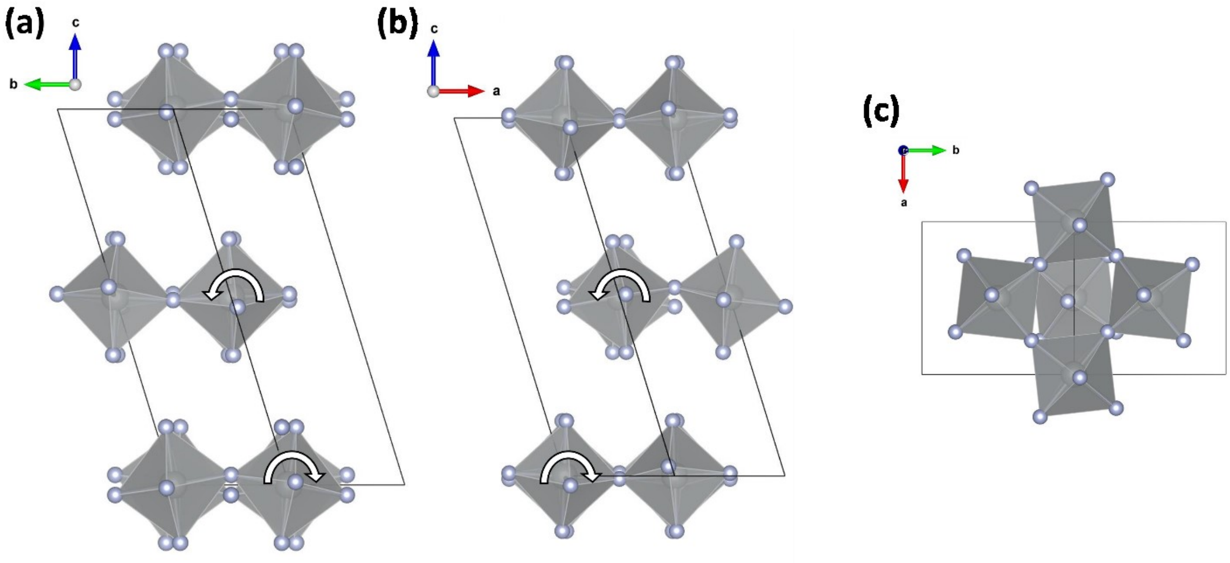

Figure S2: A schematic drawing of tilt system #15 $(\phi_1\phi_2\theta)/(\overline{\phi}_2\overline{\phi}_1\overline{\theta})$ as viewed along the (**a**) [100] direction, (**b**) [010] direction, and (**c**) [001] direction. The arrows show the direction of the ϕ tilts on the octahedra that reside at (0,0,0) and (½, ½, ½) of the parent cell. The A-site cations have been omitted for clarity.

Table S1: Tilt systems that involve a combination of ψ- and ϕ-tilts.

#	Tilts		Space group	SM3	X_3^+	X_4^+	X_2^+	Basis	Origin
	1st layer	2nd layer							
43	ψϕ0	ϕψ0	$P\bar{4}2_1c$	(0,a); (a,0)	(−b;b)	(0,0)	(0,0)	(2,0,0), (0,2,0), (0,0,1)	(1,1/2,1/4)
44	ψϕ0	$\bar{\phi}$ψ0	$P4_22_12$	(0,a); (a,0)	(0,0)	(−b;b)	(0,0)	(2,0,0), (0,2,0), (0,0,1)	(0,3/2,1/4)
45	$\phi_1\psi0$	$0\phi_20$	Pbcm	(a,0); (0,0)	(b;b)	(c;c)	(0,0)	(0,0,1), (2,0,0), (0,2,0)	(0,0,0)
46	$\psi_1\phi_10$	$\phi_2\psi_20$	$P2_12_12$	(0,a); (b,0)	(−c;c)	(−d;d)	(0,0)	(2,0,0), (0,2,0), (0,0,1)	(0,1/2,0)
47	$\phi_1\psi0$	$0\phi_2\theta$	$P2_1/c$	(a,0); (0,0)	(-b;b)	(−c;c)	(d;d)	(0,0,1), (2,0,0), (0,2,0)	(0,0,0)

Table S2: Inorganic halide RP phases from ICSD. Note that space group *Bbcm* is a non-standard setting of *Cmce*.

Compound	Temp. (K)	Space Group	Tilting		ICSD #
			Layer 1	Layer 2	
K_2MnF_4	300	*I*4/*mmm*	000	000	23183
	4.2	*I*4/*mmm*	000	000	23184
K_2CoF_4	293	*I*4/*mmm*	000	000	33522
K_2NiF_4	300	*I*4/*mmm*	000	000	73450
K_2ZnF_4	293	*I*4/*mmm*	000	000	100298
Rb_2CoF_4	293	*I*4/*mmm*	000	000	69683
Rb_2NiF_4	293	*I*4/*mmm*	000	000	69682
Rb_2MnCl_4	293	*I*4/*mmm*	000	000	1139
Rb_2CdCl_4	293	*I*4/*mmm*	000	000	51168
Cs_2CdCl_4	293	*I*4/*mmm*	000	000	16576
Rb_2MnBr_4	293	*I*4/*mmm*	000	000	8174
$(NH_4)_2MgF_4$	300	*I*4/*mmm*	000	000	82846
	20	$P2_1/c$	$\phi_1\phi_2\theta$	$\phi_2\phi_1\bar{\theta}$	82847
$Cs_2SnCl_2I_2$	293	*I*4/*mmm*	000	000	264194
$Cs_2PbCl_2I_2$	293	*I*4/*mmm*	000	000	6337
Cs_2CrCl_4	293	*I*4/*mmm*	000	000	41571
Rb_2CrCl_4	293	*Bbcm*	000	000	23394
K_2CuF_4	293	*Bbcm*	000	000	38426
$(NH_4)_2CuCl_4$	293	*Cmca*	000	000	15585
$(NH_4)_2CuF_4$	293	*Cmca*	000	000	39721
Rb_2CuCl_4	293	*Cmca*	000	000	15145

Table S3: Hybrid A_2MX_4 compositions with the Ruddlesden-Popper structure used in the analysis. The name given in the leftmost column is the name of the neutral amine, which when protonated becomes the A-site cation.

A	M^{2+}	X^{-}	Temp. (K)	Tilt Sys.	Space Group	Agrees	CCDC / ICSD #
benzylamine	Pb	Cl	93 – 423	1	*Cmc*2$_1$	No	1042742 – 1042748
benzylamine	Pb	Cl	453	1	*Cmce*	Yes	1042747
cyclobutylamine	Pb	Cl	173	11	*P*2$_1$/*c*	Yes	708567
cyclopentylamine	Pb	Cl	293	1	*Cmce*	Yes	708568
cyclopropylamine	Pb	Cl	173	11	*P*2$_1$/*c*	Yes	708566
p-flourobenzylamine	Pb	Cl	493	0	*I*4/*mmm*	Yes	1944740
m-flourobenzylamine	Pb	Cl	473	0	*I*4/*mmm*	Yes	1944742
m-flourobenzylamine	Pb	Cl	300	1	*Cmc*2$_1$	No	1944743
o-flourobenzylamine	Pb	Cl	463	0	*I*4/*mmm*	Yes	1944744
1-cyclohexylethylamine	Pb	Cl	173	14	*C*2/*c*	Yes	956552
1-cyclohexylethylamine	Pb	Cl	173	11	*P*2$_1$	No	956553
2-(ammoniomethyl)naphthalene	Pb	Br	293	1	*Cmc*2$_1$	No	1542462
benzylamine	Pb	Br	293 – 423	1	*Cmce*	Yes	1042749 – 1042752
benzylamine	Pb	Br	293	1	*Cmc*2$_1$	No	1542460
cyclobutylamine	Pb	Br	173	11	*P*2$_1$/*c*	Yes	708561
cyclohexylamine	Pb	Br	173	1	*Cmc*2$_1$	No	708563
cyclopentylamine	Pb	Br	173	11	*P*2$_1$/*c*	Yes	708562
cyclopropylamine	Pb	Br	173	11	*P*2$_1$/*c*	Yes	708560
oxan-4-amine	Pb	Br	293	1	*Cmc*2$_1$	No	1965897
2,2-difluoroethylamine	Pb	Br	295	10	*Pbca*	Yes	1938881
2,2,2-trifluoroethylamine	Pb	Br	296	1	*Pnma*	No	1938882
butylamine	Pb	Br	100	10	*Pbca*	Yes	1521054
butylamine	Pb	Br	296	21	*P*2$_1$/*a*	Yes	1903531
3-butene-1-amine	Pb	Br	296	11	*P*2$_1$/*c*	Yes	955778
3-butyne-1-amine	Pb	Br	100	11	*P*2$_1$/*c*	Yes	955776
butylamine	Pb	I	223 – 293	10	*Pbca*	Yes	665689 – 665690
benzylamine	Pb	I	293	10	*Pbca*	Yes	1525378
cyclobutylamine	Pb	I	173	11	*P*2$_1$/*c*	Yes	609993
cyclohexylamine	Pb	I	173	10	*Pbca*	Yes	609995
cyclopentylamine	Pb	I	293	11	*P*2$_1$/*c*	Yes	609994
cyclopropylamine	Pb	I	173	11	*P*2$_1$/*c*	Yes	609992

decylamine	Pb	I	243	11	$P2_1/a$	Yes	805435
decylamine	Pb	I	293	10	*Pbca*	Yes	805436
decylamine	Pb	I	343	1	*Acam*	Yes	805440
dodecylamine	Pb	I	293	10	*Pbca*	Yes	692951
dodecylamine	Pb	I	319	11	$P2_1/a$	Yes	692952
heptylamine	Pb	I	253	11	$P2_1/a$	Yes	665694
heptylamine	Pb	I	278 – 293	10	*Pbca*	Yes	805428 – 805429
hexylamine	Pb	I	173	11	$P2_1/a$	Yes	665694
hexylamine	Pb	I	293	10	*Pbca*	Yes	665693
hexadecylamine	Pb	I	293	10	*Pbca*	Yes	692955
hexadecylamine	Pb	I	341	11	$P2_1/a$	Yes	692956
nonylamine	Pb	I	223	11	$P2_1/a$	Yes	805433
nonylamine	Pb	I	293	10	*Pbca*	Yes	805434
octylamine	Pb	I	173	11	$P2_1/a$	Yes	805430
octylamine	Pb	I	293	10	*Pbca*	Yes	805431
octylamine	Pb	I	314	1	*Acam*	Yes	805432
octadecylamine	Pb	I	293	10	*Pbca*	Yes	692957
octadecylamine	Pb	I	348	11	$P2_1/a$	Yes	692958
pentylamine	Pb	I	223	11	$P2_1/a$	Yes	665691
pentylamine	Pb	I	293	11	$P2_1/a$	Yes	665692
pentylamine	Pb	I	333	10	*Pbca*	Yes	665695
tetradecylamine	Pb	I	293	10	*Pbca*	Yes	692953
tetradecylamine	Pb	I	315	11	$P2_1/a$	Yes	692954
p-fluorophenethylamine	Pb	I	100	11	$P2_1/c$	Yes	1977185
p-chlorophenethylamine	Pb	I	100	14	$C2/c$	Yes	1977186
p-bromophenethylamine	Pb	I	100	14	$C2/c$	Yes	1977187
thienylmethylamine	Pb	I	293	10	*Pbca*	Yes	187952
heptane-2-amine	Pb	I	295	11	$P2_1/c$	Yes	1863839
butan-2-amine	Pb	I	296	5	$P4_2/ncm$	Yes	1863837
pentan-2-amine	Pb	I	273	11	$P2_1/c$	Yes	1863836
isobutylamine	Pb	I	298	11	$P2_1/c$	Yes	1876240
2-phenethylamine	Sn	Cl	297	1	$Cmc2_1$	No	1962554
2-phenethylamine	Sn	Br	298	1	$Cmc2_1$	No	1962560
4-iodobutylamine	Sn	I	295.2	11	$P2_1/a$	Yes	657418
butylamine	Sn	I	128.2 – 295	10	*Pbca*	Yes	657413 – 657414, 1268428
benzylamine	Sn	I	293	10	*Pbca*	Yes	1525379

			298				1962559
hexadecylamine	Sn	I	103	10	*Pbca*	Yes	1856670
3-carboxypropylamine	Sn	I	273.2	10	*Pbca*	Yes	657419
4-(carboxy)cyclohexylmethylamine	Sn	I	293.2	1	*Cmce*	Yes	657417
cyclohexylmethanamine	Sn	I	293	11	$P2_1/c$	Yes	1503046
cyclohexylmethanamine	Sn	I	353	1	*Cmce*	Yes	1503047
dodecylamine	Sn	I	293	10	*Pbca*	Yes	223813
2-chloroethylamine	Cd	Cl	293	4	*Cmce*	Yes	1550866
butylamine	Cd	Cl	295	4	*Aema*	Yes	1200034
4-arsonoaniline	Cd	Cl	200	10	*Pbca*	Yes	1534722
cyclopropylamine	Cd	Cl	100	11	$P2_1/c$	Yes	1827265
cyclopropylamine	Cd	Cl	280	4	*Cmce*	Yes	1827266
dodecylamine	Cd	Cl	298	22	$P\bar{1}$	Yes	831018
ethylamine	Cd	Cl	178	10	*Pcab*	Yes	1147987
ethylamine	Cd	Cl	295	4	*Bmab*	Yes	1147988
isopentylamine	Cd	Cl	203	4	*Aba*2	No	1544994
isopentylamine	Cd	Cl	298	4	*Cmce*	Yes	1544993
methylamine	Cd	Cl	100	11	$P2_1/c$	Yes	1563635
methylamine	Cd	Cl	234	5	$P4_2/ncm$	Yes	1209607
methylamine	Cd	Cl	295	4	*Cmce*	Yes	1209606
2-phenethylamine	Cd	Cl	293	4	*C*2*ce*	No	964405
2-phenethylamine	Cd	Cl	298	10	*Pbca*	Yes	129024
propylamine	Cd	Cl	165	10	*Pbca*	Yes	1237918
propylamine	Cd	Cl	295	4	*Aema*	Yes	1237917
cyclohexylamine	Cd	Cl	298	1	$Cmc2_1$	No	998556
p-nitroaniline	Cd	Cl	298	10	*Pbca*	Yes	1312594
isopentylamine	Cd	Br	298	4	*Cmce*	Yes	1544994
isobutylamine	Cd	Br	93	4	*Aea*2	No	1495225
isobutylamine	Cd	Br	193 – 283	4	*Cmce*	Yes	1495222 – 1495224
2,2-difluoroethylamine	Cd	Br	100	10	*Pbca*	Yes	1960260
cyclohexylamine	Cd	Br	300	1	$Cmc2_1$	No	1822417
2-chloroethylamine	Cd	Br	293	4	*Aea*2	No	1550869
2-phenethylamine	Mn	Cl	100	10	$Pca2_1$	No	1583818
2-phenethylamine	Mn	Cl	296	10	*Pbca*	Yes	258590
2-phenethylamine	Mn	Cl	430	0	*I*4/*mmm*	Yes	1583818
undecylamine	Mn	Cl	293	11	$P2_1/c$	Yes	969103
octylamine	Mn	Cl	295	11	$P2_1/a$	Yes	1137718
ethylamine	Mn	Cl	126	10	*Pbca*	Yes	1148005

methylamine	Mn	Cl	188	5	$P4_2/ncm$	Yes	110654
methylamine	Mn	Cl	295	4	*Aema*	Yes	1209613
propylamine	Mn	Cl	182	4	*Abma*	Yes	1227773
6-chloro-5-methylpyridin-3-amine	Cu	Cl	296	11	$P2_1/c$	Yes	1921597
ethylamine	Cu	Cl	110 – 273	10	*Pbca*	Yes	1955780 – 1955781
butylamine	Cu	Cl	188 – 223	11	$P2_1/c$	Yes	1955785 – 1055787
pentylamine	Cu	Cl	300	6	*Pccn*	Yes	1955788
pentylamine	Cu	Cl	250	22	$P\bar{1}$	Yes	1955789
propylamine	Cu	Cl	273	10	*Pbca*	Yes	1955783
11-ammonioundecanoic acid	Cu	Cl	295	22	$P\bar{1}$	Yes	1102856
aniline	Cu	Cl	295	11	$P2_1/c$	Yes	1102978
heptylamine	Cu	Cl	200	22	$P\bar{1}$	Yes	1955790
methylamine	Cu	Cl	100 – 293	11	$P2_1/a$	Yes	1319987 – 110695
3-carboxypropanamine	Cu	Cl	293	11	$P2_1/c$	Yes	239070
2-phenethylamine	Cu	Cl	100	10	*Pbca*	Yes	751958
2-phenethylamine	Cu	Cl	373	1	*Cmce*	Yes	892624
p-fluoroaniline	Cu	Cl	293	11	$P2_1/c$	Yes	781208
decylamine	Cu	Cl	298	22	$P\bar{1}$	Yes	833345
undecylamine	Cu	Cl	298	22	$P\bar{1}$	Yes	833346
isobutylamine	Cu	Cl	90	11	$P2_1/c$	Yes	874688
p-cyanoaniline	Cu	Cl	295	22	$P\bar{1}$	Yes	1229035
p-chloroaniline	Cu	Cl	295	11	$P2_1/c$	Yes	1229036
p-nitroaniline	Cu	Cl	295	10	*Pbca*	Yes	1229037
4-arsonoaniline	Cu	Cl	200	10	*Pbca*	Yes	1534721
2-chloroethylamine	Cu	Cl	296	11	$P2_1/c$	Yes	1913462
p-biphenylamine	Cu	Cl	100	10	*Pbca*	Yes	237779
cyclohexylamine	Cu	Br	298	11	$P2_1/c$	Yes	293067
p-chloroaniline	Cu	Br	296	10	*Pbca*	Yes	1278772
methylamine	Fe	Cl	300	6	*Pccn*	Yes	989655
methylamine	Fe	Cl	340	0	*I4/mmm*	Yes	989650
ethylamine	Fe	Cl	150	10	*Pbca*	Yes	1485141
ethylamine	Fe	Cl	296	6	*Pccn*	Yes	1485145
ethylamine	Fe	Cl	350	5	$P4_2/ncm$	Yes	1485146
ethylamine	Fe	Cl	383	0	*I4/mmm*	Yes	1485144
propylamine	Fe	Cl	293	4	*Cmca*	Yes	721801
benzylamine	Cr	Br	295	10	*Pcab*	Yes	1280512

Table S4: Hybrid A_2MX_4 phases that were not included in the analysis either because they possess layer shift factors different from 0.5 *a* + 0.5 *b* characteristic of the RP structure type, or because the *.cif file had flags that calls into question it's accuracy, either unresolved level A or B alerts or high R-factors.

A	M^{2+}	X^{-}	Temp. (K)	Tilt Sys.	Space Group	CCDC #	Layer Shift or *.cif issue
2-(2-ammonioethyl)naphthalene	Pb	Br	293	1	*P*1	1542464	*.cif issue
2-fluoro-ethylamine	Pb	Br	296	1	*Pnma*	1938883	0.42 + 0.50
N-methyl-3-fluoro-cyclohexylamine	Pb	Br	296	10	*Pbcn*	1845548	0 + 0.17
N-methyl-3-fluoro-cyclohexylamine	Pb	Br	413	1	*Cmcm*	1852626	0 + 0.15
piperidine	Pb	Br	210	?	*Pnma*	1962914	*.cif issue
1-2-napthylmethylamine	Pb	Cl	293	1	*Pbam*	120686	0 + 0
cyclohexylamine	Pb	Cl	173	1	$P2_1/m$	708569	0.21 + 0.23
2-phenethylamine	Pb	Cl	293	22	$P\bar{1}$	1209430	0.25 + 0.50
propylamine	Pb	Cl	293	1	*Pnma*	1181686	0.19 + 0.50
p-fluoro-benzylamine	Pb	Cl	300	1	*Pnma*	1944739	0.17 + 0.50
m-fluoro-benzylamine	Pb	Cl	300	11	$P2_1/c$	1944741	0 + 0.15
2-fluoro-ethylamine	Pb	Cl	173	1	*Pnma*	1479690	0.16 + 0.50
4-methyl-benzylamine	Pb	Cl	293	11	$P2_1/c$	141193	0.07 + 0
piperidine	Pb	Cl	373	5	*C*2/*c*	1962913	*.cif issue
2-(1-cyclohexenyl)ethylamine	Pb	I	173	22	$P\bar{1}$	616101	0 + 0.50
2-(2-ammonioethyl)napthalene	Pb	I	293	22	*Pn*	1542463	0.25 + 0.50
2-phenethylamine	Pb	I	100	11	*Cc*	1841681	0.05 + 0.50
2-phenethylamine	Pb	I	293	25	*P*-1	1977183	*.cif issue
p-chloro-aniline	Pb	I	120	11	$P2_1/c$	747016	0 + 0.17
2-phenethylamine	Sn	Br	293	12	$P\bar{1}$	1004372	0.25 + 0.50
butylamine	Ge	I	295	10	*Pcmn*	TECDOU	*.cif issue
ethynamine	Cd	Cl	173	10	*C*2/*c*	1203501	0 + 0.25
undecylamine	Cd	Cl	298	14	*C*2/*c*	847667	0.50 + 0.45
pentylamine	Cd	Cl	173	4	*C*2/*m*	1565821	0.50 + 0.04
p-tolylamine	Cd	Cl	289	1	*Pnma*	852580	0.39 + 0.50
2-phenethylamine	Mn	Cl	385	0	*Aea*2	1583817	*.cif issue
ethylamine	Fe	Cl	10	4	*C*2/*c*	1485137	*.cif issue
ethylamine	Fe	Cl	300	10	*Pbca*	1494749	*.cif issue

dodecylamine	Cu	Cl	298	22	$P\bar{1}$	777513	0 + 0
hexadecylamine	Cu	Cl	298	14	*Cc*	780691	0.50 + 0.45
tetradecylamine	Cu	Cl	298	14	*C*2/*c*	780690	0.50 + 0.40
octylamine	Cu	Cl	296	11	$P\bar{1}$	805760	0 + 0
3-carboxypropanamine	Cu	Cl	295	14	*C*2/*c*	1108061	0 + 0
4-phenylbutylamine	Cu	Cl	120	22	$P2_1$	1441233	0 + 0
4-phenylbutylamine	Cu	Cl	350	0	*P*4/*mbm*	1441232	0 + 0
4-phenylbutylamine	Cu	Cl	293	11	$P2_1/a$	1441231	0 + 0
propylamine	Cu	Cl	110	10	$P2_12_12_1$	1955782	*.cif issue
3-carboxypropanamine	Cu	Br	296	14	*C*2/*c*	1121167	0 + 0

Figure S3: Bond valence sums for the terminal and bridging halides across a series of Pb-containing RP phases.

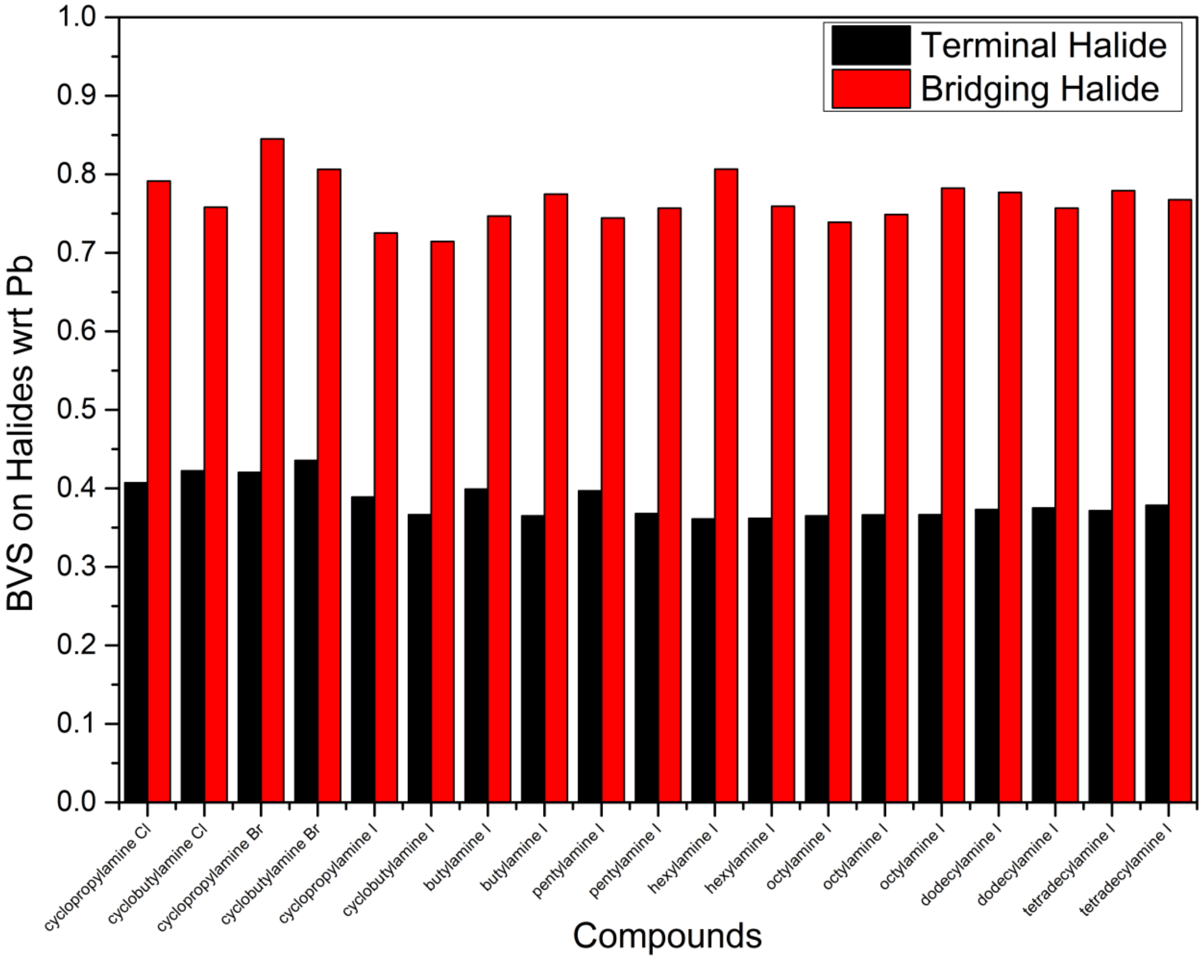

Table S5: Polar compositions that do not match symmetry analysis.

A	M^{2+}	X^{-}	Temp. (K)	Tilt Sys.	Space Group	CCDC / ICSD #
benzylamine	Pb	Cl	93 – 423	1	$Cmc2_1$	1042742 – 1042748
m-flourobenzylamine	Pb	Cl	300	1	$Cmc2_1$	1944743
1-cyclohexylethylamine	Pb	Cl	173	11	$P2_1$	956553
2-(ammoniomethyl)naphthalene	Pb	Br	293	1	$Cmc2_1$	1542462
benzylamine	Pb	Br	293	1	$Cmc2_1$	1542460
cyclohexylamine	Pb	Br	173	1	$Cmc2_1$	708563
oxan-4-amine	Pb	Br	293	1	$Cmc2_1$	1965897
2-phenethylamine	Sn	Cl	297	1	$Cmc2_1$	1962554
2-phenethylamine	Sn	Br	298	1	$Cmc2_1$	1962560
isopentylamine	Cd	Cl	203	4	$Aba2$	1544994
2-phenethylamine	Cd	Cl	293	4	$C2ce$	964405
cyclohexylamine	Cd	Cl	298	1	$Cmc2_1$	998556
isobutylamine	Cd	Br	93	4	$Aea2$	1495225
cyclohexylamine	Cd	Br	300	1	$Cmc2_1$	1822417
2-chloroethylamine	Cd	Br	293	4	$Aea2$	1550869
2-phenethylamine	Mn	Cl	100	10	$Pca2_1$	1583818
